# Age-Dependent Maturation of iPSC-CMs Leads to the Enhanced Compartmentation of β_2_AR-cAMP Signalling

**DOI:** 10.3390/cells9102275

**Published:** 2020-10-12

**Authors:** Alveera Hasan, Neda Mohammadi, Aisha Nawaz, Thusharika Kodagoda, Ivan Diakonov, Sian E. Harding, Julia Gorelik

**Affiliations:** Cardiac Section, National Heart and Lung Institute (NHLI), Faculty of Medicine, Imperial College London, Hammersmith Campus, Du Cane Road, London W12 0NN, UK; alveera.hasan13@imperial.ac.uk (A.H.); neda.mohammadi18@imperial.ac.uk (N.M.); aisha.nawaz@imperial.ac.uk (A.N.); t.kodagoda@imperial.ac.uk (T.K.); i.diakonov@imperial.ac.uk (I.D.); s.harding@imperail.ac.uk (S.E.H.)

**Keywords:** cAMP, β-adrenergic receptors, phosphodiesterase, caveolae, FRET, iPSC-cardiomyocytes

## Abstract

The ability to differentiate induced-pluripotent stem cells into cardiomyocytes (iPSC-CMs) has opened up novel avenues for potential cardiac therapies. However, iPSC-CMs exhibit a range of somewhat immature functional properties. This study explored the development of the beta-adrenergic receptor (βAR) pathway, which is crucial in regulating contraction and signifying the health and maturity of myocytes. We explored the compartmentation of β_2_AR-signalling and phosphodiesterases (PDEs) in caveolae, as functional nanodomains supporting the mature phenotype. Förster Resonance Energy Transfer (FRET) microscopy was used to study the cyclic adenosine monophosphate (cAMP) levels in iPSC-CMs at day 30, 60, and 90 following βAR subtype-specific stimulation. Subsequently, the PDE2, PDE3, and PDE4 activity was investigated using specific inhibitors. Cells were treated with methyl-β-cyclodextrin (MβCD) to remove cholesterol as a method of decompartmentalising β_2_AR. As iPSC-CMs mature with a prolonged culture time, the caveolae density is increased, leading to a reduction in the overall cytoplasmic cAMP signal stimulated through β_2_AR (but not β_1_AR). Pan-phosphodiesterase inhibition or caveolae depletion leads to an increase in the β_2_AR-stimulated cytoplasmic cAMP. Moreover, with time in culture, the increase in the βAR-dependent cytoplasmic cAMP becomes more sensitive to cholesterol removal. The regulation of the β_2_AR response by PDE2 and 4 is similarly increased with the time in culture. We conclude that both the β_2_AR and PDEs are restricted to the caveolae nanodomains, and thereby exhibit a tighter spatial restriction over the cAMP signal in late-stage compared to early iPSC-CMs.

## 1. Introduction

Over the past decade, tremendous progress has been made in the development of pluripotent stem cell-derived cardiomyocytes as a source of healthy cells, not only for in vitro drug testing and disease modelling but also to fulfil a greater promise for cardiac regeneration. However, the major challenge is in overcoming the immaturity of pluripotent stem cell-derived cardiomyocytes. One of the key characteristics and indicators of cardiomyocyte maturity is their well-defined sarcomeres. The intricate alignment of cardiac contractile proteins is required for achieving stronger, more efficient cardiomyocyte contractions to meet the workload demand. Although induced pluripotent stem cell-derived cardiomyocytes (iPSC-CMs) are known to express sarcomeric proteins and cardiac-specific transcription factors [1,2], they still display underdeveloped cell morphology, indicating a poor or limited organisation of intracellular compartments [3]. Multiple methods of improving iPSC differentiation protocols have been explored as a method of overcoming structural immaturity [4]; however, the “rod/barrel”-like shape, Ca^2+^ -handling, and biochemical characteristics exhibited by mature adult cardiomyocytes were still not achieved through differentiation protocols alone.

The underdevelopment of iPSC-CMs may underlie the immaturity in their physiological functions, such as smaller transient amplitudes [5] and slowed excitation-contractions kinetics, contractile force, and relaxation [3,6,7]. It is established that Ca^2+^-handling properties, adrenergic signalling, and contractility rely on distinct cardiac microdomains. For example, healthy cardiomyocytes possess highly intricate membrane structures, such as T-tubular networks and caveolae components [8]. These form functional domains by representing sites where receptors and their associated signalling molecules are clustered, thus ensuring effective signal processing and transduction [9]. Caveolae have been indicated as precursors of T-tubules in early myocytes, hence caveolae complexes can partially fulfil some of the required signalosome functions in developing cardiomyocytes [10]. Despite evidence of membrane caveolae being present in iPSC-CMS, their progression into deeper T-tubular invaginations is not achieved in monolayer cultures [11]. 

Several studies have shown that the prolonging culture time of iPSC-CMs to 120 days after the onset of cardiac differentiation leads to improvements in morphology and cell function, such as multinucleation, cell size, sarcomere length, and organisation [12,13]. In a recent study, Jung et al. investigated the expression and functional developments of βAR signalling and progressed to report the evolution of the signalling components upon prolonging the iPSC-CM culture period [14]. They found that β_2_ARs are the dominant receptor subtype and are the primary source of cAMP/PKA signalling in “early” cardiomyocytes. Interestingly, increasing the iPSC-CMs culture time from 30 to 60 days led to the β_1_AR expression increasing significantly and the β_1_AR -dependent cAMP production increasing nine-fold, thus representing a more “adult-like” phenotype. Furthermore, they detected an increase in the chronotropic response to βAR stimulation with a prolonged time after differentiation, with a 40% higher beating rate at D90. Furthermore, they also highlight an increased level of the caveolin-3 (Cav3) gene and protein expression in aged iPSC-CMs.

The catecholamine stimulation of βAR leads to the activation of adenylate cyclase and the consequent rise in cytosolic cAMP and the phosphorylation of downstream protein kinases, thus triggering the onset of myocyte contraction [15]. It has been previously shown that treating iPSC-CMs with forskolin, a direct activator of adenylate cyclase, and with isobutylmethylxanthine (IBMX), a phosphodiesterase inhibitor, exerts a positive chronotropic effect, suggesting that the complexes required in this signalling pathway are already present early in human cardiomyocytic differentiation [16]. In addition, there is growing evidence suggesting that cAMP in healthy adult cardiomyocytes is confined in discrete subcellular compartments such as the T-tubules and caveolae. In part, it occurs through phosphodiesterases (PDEs), enzymes which degrade cyclic nucleotides [17,18,19] and can act as functional barriers by preventing its free diffusion throughout the cell [20]. It has been shown that blocking all PDEs after βAR stimulation leads to changes in the physiological response due to disruption in the spatial and temporal regulation of cAMP [21,22]. This highlights the importance of the extensive assortment of PDEs in modulating βAR-dependent cAMP and indicating well-regulated/developed cAMP handling. 

We hypothesise that assessing βAR–dependent cAMP signalling responses can prove to be an effective method for tracking the functional maturation of iPSC-CMs. To further develop this idea, we aim to define the role of the age-dependent maturation of iPSC-CMs in the development of caveolae structures and functional signalosomes, thus investigating its influence on β_2_AR-cAMP signalling. Furthermore, we aim to understand the effect of prolonged culture time on the functional activity of specific PDEs, thus depicting the regulation of the βAR-dependent cAMP within these microdomains. 

## 2. Materials and Methods

### 2.1. Generation and Differentiation of iPSC-CMs

Human iPSCs cell line IMR-90, derived from human foetal lung fibroblasts (Wicell, Madison, WI, USA), was differentiated into hiPSC-CMs using a monolayer differentiation protocol [23,24]. Briefly, hiPSCs were plated onto plastic culture plates coated with Matrigel (Thermo-Fisher Scientific, Loughborough, United Kingdom) and maintained in E8 media (StemCell Technologies UK, Cambridge, United Kingdom). At a 90% confluency, they were treated with 8 µM of CHIR99021 (Bio-Techne Ltd., Abingdon, United Kingdom) to initiate differentiation (Day0). On D2, the media were replaced with RPMI plus B27 without insulin (RB-) (Thermo-Fisher Scientific, Loughborough, United Kingdom). On D3, cells were treated with 5 µM C59 (Bio-Techne Ltd., Abingdon, United Kingdom) in RB- and then returned back to RB- on D5. On D5, metabolic selection was initiated (RPMI-1640 without glucose) to remove non-cardiomyocytes and obtain a >95% cardiomyocyte purity. On D15, the iPSC-CMs were replated on plastic-bottomed culture dishes and cultured in RPMI + B27 medium every other day until they were ready for experiments. At the required age (D30, D60, D90), the cells were dissociated and plated at the required density on Fibronectin-coated glass-bottom MatTek dishes (MatTek Corporation, Ashland, MA, USA) for experimental use. 

Another cell line used in this study was iCell^®^ Cardiomyocytes obtained from FUJIFILM Cellular Dynamics, Inc., Madison, WI, USA. These cells were purchased frozen and at various states of maturity. The 1.5 mL cyropreserved single-cell suspension vials were thawed, seeded, and cultured following the user guide protocols. They were plated on 0.1% gelatin and were cultured for the recommended 10 days to reach maximum stability, equivalent to D30 cells.

### 2.2. iPSC-CM Transfection

iPSC-CMs were transfected with the cAMP-specific FRET sensor plasmid Epac-S^H74^ [25], and/or with the CAV3 plasmid for caveolin-3 overexpression (Addgene, Watertown, MA, USA). The plasmid DNA (500 ng) and Lipofectamine3000 reagent were diluted in OptiMEM (both Thermo-Fisher Scientific, Loughborough, United Kingdom) separately and incubated for 5 min before combining and incubating for 20 min, and then they were added to cells 48 h prior to experimentation. 

### 2.3. Transmission Electron Microscopy

The caveolae number was assessed by TEM in control and methyl-β-cyclodextrin (MβCD) (Merck Life Science UK Ltd, Gillingham, United Kingdom) treated. Culture medium was removed from the cell culture dishes; cells were rinsed with PBS and then fixed in 2.5% gluteraldehyde in cacodylate buffer. The sample was then post-fixed with osmium tetroxide, processed according to the standard protocol, and embedded in Araldite. Sections were cut from the hardened sample and stained with methylene blue, then trimmed around the areas containing the cells. Then, 0.1 μm sections were obtained and stained with uranyl acetate and lead citrate to be visualised with a transmission electron microscope. To determine the effect of depleting caveolae, the cells were treated with MβCD and incubated at 37 °C for 1 h before fixation. Cells were also transfected with CAV3 for protein overexpression at D30 as detailed above. From each TEM sample preparation, typically, 10 cells were imaged. From each cell, 10 frames were recorded at a 20,000× magnification, and the average number of caveolae per µm of membrane was calculated.

### 2.4. FRET Microscopy

The FRET imaging system in our laboratory consisted of a Nikon Eclipse TE200-U microscope (Nikon Instruments Europe BV, Amsterdam, Netherlands), an LED light source (Cairn Research, Faversham, United Kingdom), a filter cube with EX436/20 excitation filter and DM455 dichroic mirror (Chroma Technology Corporation, Bellows Falls, VT, USA), a 60× objective lens, and a Quadview beam splitter equipped with 535/40 and 480/30 emission filters to separate the cell fluorescence into YFP and CFP channels (Teledyne Photometrics, Tucson, AZ, USA). Time-lapse imaging was executed with a Hamamatsu ORCA-Flash4.0 camera (Hamamatsu Photonics UK Ltd, Welwyn Garden City, United Kingdom), with a frame every 6 seconds. The image acquisition and analysis by quantifying the relative ratio of YFP to CFP image intensity in the regions of interest (ROI) was performed using a custom-made plug-in to Icy freeware (http://icy.bioimageanalysis.org/) using the Micro-Manager1.4 (http://micro-manager.org) software package [26].

Experiments were performed in a FRET buffer (NaCl 144 mM, HEPES 10 mM, MgCl2 1 mM, KCl 5 mM – pH7.4). Culture dishes with a maximum volume of 2.5 mL were utilised. To determine the β_2_AR response, β_1_AR was blocked by incubating cells in CGP20712A (CGP) (Thermo-Fisher Scientific, Loughborough, United Kingdom) (100 nM) for 10 min. To determine the β_1_AR response, β_2_AR was blocked by incubating cells in ICI 118,551 (ICI) (Bio-Techne Ltd., Abingdon, United Kingdom) (50 nM) for 10 mins. As the FRET imaging was running, isoprenaline (ISO) (30 nM) was added to the bath before the addition of NKH477 (NKH) (Thermo-Fisher Scientific, Loughborough, United Kingdom) (10 μM) to fully stimulate all the adenylate cyclase activity. To assess PDE activity EHNA (10 μM), Cilostamide (CILO) (10 μM), Rolipram (ROLI) (all tree purchased from Bio-Techne Ltd., Abingdon, United Kingdom) (10 μM) were used. For non-selective PDE inhibition, IBMX (Thermo-Fisher Scientific, Loughborough, United Kingdom) (100 μM) was used. The ratio of the Venus to Turquoise signal was calculated and normalised to the maximum FRET response given by adenylate cyclase activator NKH477. For the disruption of caveolae, 1–2 mM Methyl-β-cyclodextrin (MβCD) (Thermo-Fisher Scientific, Loughborough, United Kingdom) was added for 1 h prior to experimentation. The percentage change in the FRET ratio was calculated as (Rm-Ro)/Ro x100 where Rm is ratio after MβCD, Ro is ratio before MβCD.

### 2.5. Immunocytochemistry

Cells were fixed for 10 min with 3.7% formaldehyde solution (F1635, Thermo-Fisher Scientific, Loughborough, United Kingdom) in PBS then washed with PBS. Cells were permeabilised with 0.5% Triton X-100 at room temperature for 15 min and then incubated in blocking buffer (PBS + 10% FBS) for 30 min. Samples were treated with anti-cav3 primary antibody (mouse) (1:100) diluted in blocking buffer for 2 h at room temperature. After washing with PBS, the cells were incubated in secondary antibody (donkey) and DAPI for 1 h. Cells were rinsed with PBS then mounted with Vectashield media before imaging by confocal microscopy.

### 2.6. RT-QPCR

RNA was extracted using the RNeasy Mini kit (Qiagen, Manchester, United Kingdom) from cultured hiPSC-CMs according to the manufacturer’s guidelines. The total mRNA concentration was measured using the NanoDrop UV-Vis Spectrophotometer (Thermo-Fisher Scientific, Loughborough, United Kingdom). The Qiagen OneStep RT-PCR kit was used to perform RT-QPCR (Qiagen, Manchester, United Kingdom). Primer sequences are given in Supplementary Table S1. 

### 2.7. Statistical Analysis

The statistical tests conducted to analyse data obtained from experimental work were the Student’s T-test or the one-way analysis of variance (ANOVA), which were performed using GraphPad Prism 5. The statistical significance was set at *p* < 0.05 and data were reported with ±SEM (standard error of the mean). Data on graphs are given as mean ± SEM. For the percentage change data in Figure 5, the SD was calculated as described [27].

## 3. Results

### 3.1. The Effect of Increased Time on β_1_ar and β_2_ar Gene Expression and Camp Output upon Isoprenaline Stimulation

**Figure 1:**
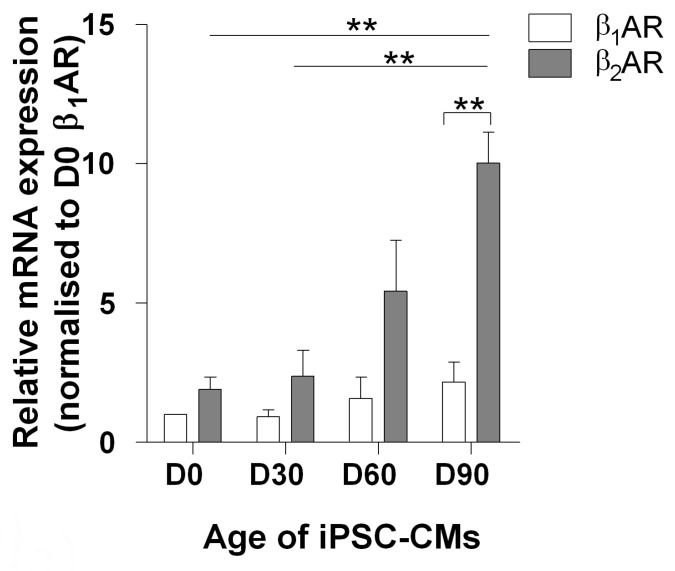
Stimulation of βAR with isoprenaline induces dose-dependent cAMP release. Differential ADRB1 and ADRB2 gene expression in iPSCs (D0) and iPSC-CMs after 30, 60, and 90 days of differentiation. n/N = 3/3; n/N = replicates/batches. Data shown as mean ± SEM, ** *p* < 0.01 between marked groups; β_2_AR D0 vs. β_2_AR D90, β_2_AR D30 vs. β_2_AR D90, and β_1_AR D90 vs. β_2_AR D90 (1-way ANOVA with Tukey’s post hoc test).

To analyse the functional maturation of βAR-cAMP signalling, we extended the culture period of hiPSCs after cardiac differentiation for up to 90 days. We investigated the maturation of the βAR-related pathways. Firstly, we assessed the mRNA expression levels of β_1_AR and β_2_AR in iPSC-CMs as the respective ages with RT-qPCR (Figure 1). We observed slightly greater β_2_AR expression levels in comparison to β_1_AR in early (D30) cardiomyocytes, which are similar to foetal and neonatal CMs [28]. With time, the mRNA level of both receptors increased. By D90, there was a two-fold increase in the β_1_AR expression (although not statistically significant), and a significant (five times greater) increase in the β_2_AR expression levels. Secondly, we investigated the functional responses of β_1_AR and β_2_AR in iPSC-CMs at D30 and D90. 

### 3.2. Age-Dependent Maturation of Ipsc-CMS Increases Caveolae Density

**Figure 2:**
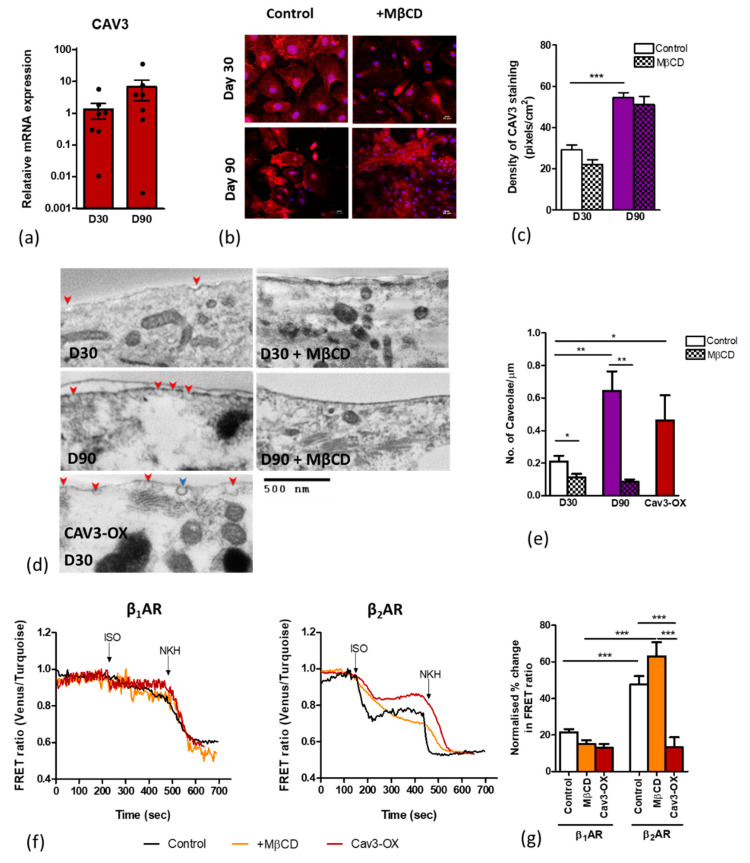
Age-dependent maturation specifically increases the caveolae content. (**a**) CAV3 gene expression in prolonged culture (n/N = 7/3). (**b**) Immunofluorescence staining for caveolin-3 (red) at D30 and D90. Nuclei are stained with DAPI (blue); (**c**) quantification of Cav3 protein density shows a significant increase from D30 (n/N = 19/3) to D90 (n/N = 13/3) *** *p* < 0.001 between D30 and D90. (**d**) TEM images showing surface caveolae (indicated with red arrows; clathrin pit is indicated with blue arrow) in control ± MβCD treatment, aged iPSC-CMs to D90 ± MβCD, and in Cav3-OX iPSC-CMs; (**e**) quantification of the caveolae density on the cell membrane in D30 control (n/N= 45/2), D30 MβCD-treated (n/N = 37/2), D90 control (n/N = 10/2), D90 MβCD (n/N= 13/2), and Cav3-OX (n/N = 12/1) cells. Data are presented as mean ± SEM. Significance was determined by one-way ANOVA with Bonferroni’s post-hoc test. * *p* < 0.05, ** *p* < 0.01; (**f**) representative FRET traces in control (black), MβCD-treated (orange), and Cav3-OX (red) iPSC-CMs (D30) upon either β_1_AR or β_2_AR stimulation with isoprenaline (30 µM) in the presence of a specific blocker (CGP (100 nM) or ICI (50 nM), respectively) and, subsequently, the addition of NKH (10 µM) for adenyl cyclase stimulation. (**g**) The percentage change in the FRET ratio upon β_1_AR stimulation in control (n/N = 97/11), +MβCD (n = 62/11), and Cav3-OX (n = 6/2); and β_2_AR stimulation in control (n/N = 38/4), +MβCD (n = 28/3), and Cav3-OX (n/N = 9/2). Percentage change was calculated and normalised to the maximal cAMP released by the NKH477 stimulation. Data are presented as mean ± SEM. Significance was determined by one-way ANOVA with Bonferroni’s post-hoc test. *** *p* < 0.001. n/N = cells/batches.

We next investigated the caveolae content in early cells at D30 and aged cells at D90. A Cav3 expression analysis revealed an increase in the relative mRNA expression from 1.32 at D30 to 6.72 at D90 (*p* = 0.23) (Figure 2a). A significant increase in the Cav3 staining density was also detected upon aging iPSC-CMs. Treatment with MβCD, (which removes cholesterol from the lipid membranes) did not significantly change the Cav3 staining density in either control or aged cells, as seen with immunofluorescence (Figure 2b,c). We conducted a TEM investigation of the membrane surface caveolae at D30 and D90 in control, caveolae-depleted (MβCD-treated), and Cav3-overexpresing cells. The quantification of caveolae density in the TEM images showed a significantly increased number of membrane caveolae at D90 as compared with D30 (average of 0.64 caveolae per µm versus 0.21 per µm, respectively). Similarly, the overexpression of Cav3 in D30 iPSC-CMs significantly increased the membrane caveolae density. Treatment with MβCD significantly reduced the membrane caveolae (Figure 2d,e).

### 3.3. Caveolar Microdomains Compartmentalise β_2_AR and Localise its Associated cAMP Signals

We then used FRET microscopy to assess whether the caveolae content correlates with the compartmentation of βAR-cAMP signalling. To assess the differential response of the β_1_AR and β_2_AR-dependent cAMP output, we used CGP and ICI, respectively, as selective βAR blockers prior to receptor stimulation with isoprenaline, and AC activation with forskolin analog, NKH477, as the maximal saturation response. MβCD was used to deplete the membrane caveolae. FRET analysis revealed that selective β_2_AR stimulation results in a 47.69% rise in the cytoplasmic cAMP output, a significantly greater increase in the cAMP output than that following β_1_AR stimulation, 21.56% (Figure 2f,g). Interestingly, the caveolae depletion with MβCD treatment only elevated the cytoplasmic β_2_AR but not β_1_AR-dependent cAMP response, indicating that there had been some degree of β_2_AR compartmentation. Conversely, enhancing the caveolae content through overexpressing Cav3 significantly reduced the amount of detectable cytosolic cAMP from β_2_AR stimulation to 13.34% (Figure 2f,g). 

Interestingly, another iPS-CM cell line (iCell) that we have tested exhibited even an more potent increase in both β_2_AR-dependent and β_2_AR-dependent cAMP release upon MβCD treatment (Figure S1).

### 3.4. Aging Ipsc-CMS Reduces Detectable β_2_ar-Dependent Camp

**Figure 3:**
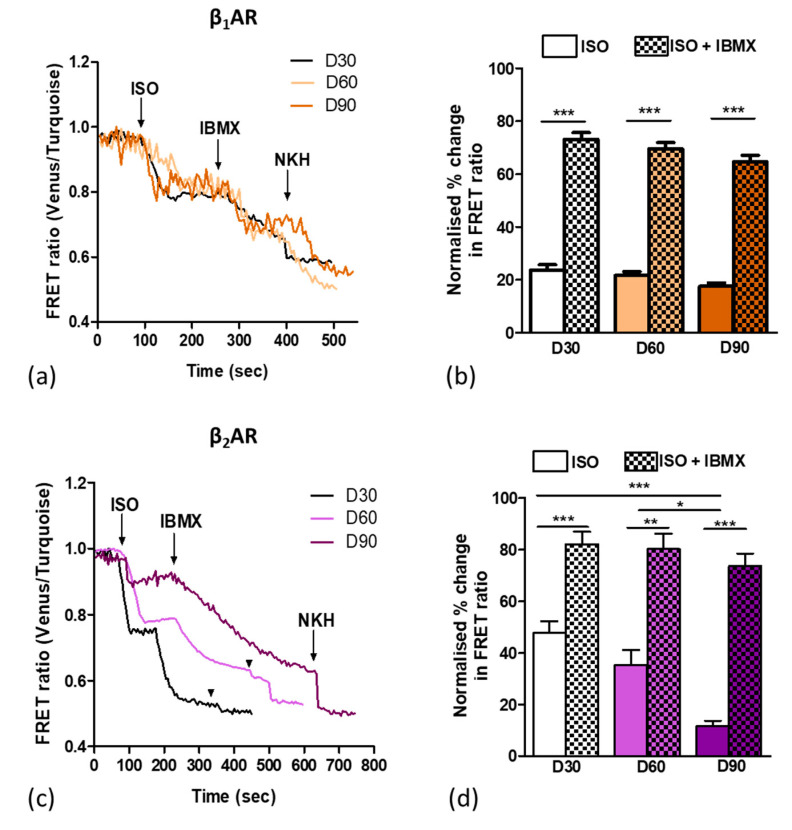
Time-dependent maturation leads to reduced cytosolic β_2_AR-dependent cAMP signals. (**a**) Representative FRET traces of D30, D60, and D90 iPSC-CMs upon β_1_AR stimulation with ISO in the presence of β_2_AR blocker, then the addition of IBMX for PDE inhibition and lastly the addition of NKH477 for the subsequent activation of AC. (**b**) Percentage change in the FRET ratio normalised to the maximal cAMP released by NKH stimulation D30 (n/N= 97/11), D60 (n/N= 158/11), and D90 (n/N= 121/8) for β_1_AR responses. (**c**) Representative FRET traces of D30, D60, and D90 iPSC-CMs upon β_2_AR stimulation with ISO in the presence of β_1_AR blocker, PDE inhibition with IBMX, and the subsequent activation of AC with NKH477. (**d**) Percentage change in the FRET ratio normalised to the maximal cAMP released by NKH477 stimulation at D30 (n/N = 38/4), D60 (n/N = 15/3), and D90 (n/N = 90/5) for β_2_AR responses. Data are presented as mean ± SEM. Significance was determined by one-way ANOVA with Bonferroni’s post-hoc test. * *p* < 0.05, ** *p* < 0.01, *** *p* < 0.001. n/N = cells/batches.

As aged cells displayed a greater abundance of caveolae components, we investigated the compartmentation of β_2_AR within caveolae by conducting βAR-cAMP FRET experiments, as iPSC-CMs are aged from D30 to D90. We investigated the basal level of cAMP release upon the βAR-dependent stimulation with ISO. We found that the β_1_AR-cAMP output remains unaltered from D30 to D90 (Figure 3a,b). Interestingly, however, the β_2_AR-cAMP is significantly reduced from a 47.69% cytoplasmic cAMP output at D30 to 11.54% at D90 (*p* < 0.001) (Figure 3c,d). With the pan-inhibition of all PDEs with a non-selective PDE inhibitor, IBMX, we observe a consistent increase in cAMP to a similar value at all time points after both β_1_AR and β_2_AR stimulation. This is consistent with cAMP production within a compartment separate from the main cytosol, spatially constrained by phosphodiesterase activity. While the β_1_AR-cAMP does not change from 30 to 90 days, the β_2_AR-cAMP becomes increasingly compartmentalised. 

### 3.5. Role of Pdes in Compartmentation of βAr-Dependent Camp Release

We then assessed the relative contributions of three PDEs towards the selective cAMP degradation post-stimulation of β_1_AR or β_2_AR with isoprenaline. The role of PDE2, 3, and 4 in iPSC-CMs was assessed through selective inhibition using EHNA, Cilostamide, and Rolipram, respectively. FRET analysis revealed significant differences in cAMP handling by β_1_AR and β_2_AR stimulation.

**Figure 4:**
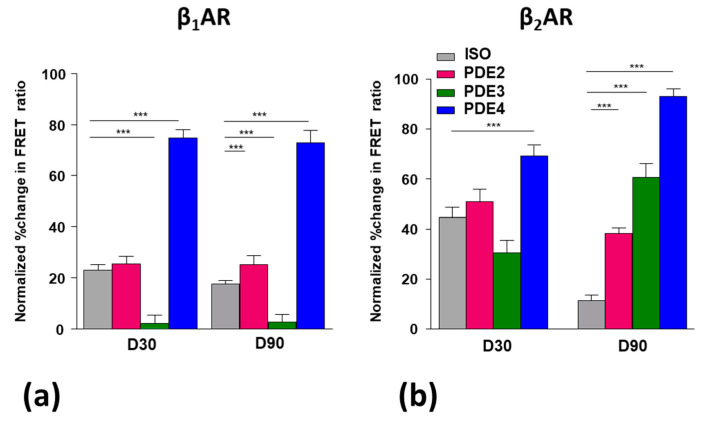
Influence of PDE2, 3, and 4 inhibition on the cAMP activity in D30 and D90 iPSC-CMs after stimulation with isoprenaline. (**a**) Normalised FRET ratio in iPSC-CMs at D30 following either β_1_AR or β_2_AR stimulation with isoprenaline (30 µM) in the presence of ICI (50 nM) (97/11) with subsequent PDE inhibition; in iPSC-CMs at D30 PDE2 (26/5), PDE3 (33/2), and PDE4 (38/6) and D90 with ISO (121/7) and the subsequent inhibition of PDE2 (48/3), PDE3 (51/2), and PDE4 (36/3); (**b**) normalised FRET ratio in iPSC-CMs at D30 following either β_1_AR or β_2_AR stimulation with isoprenaline (30 nM) in the presence of CGP (100 nM) (38/4), with the subsequent inhibition of PDE2 (10/3), PDE3 (22/7), and PDE4 (33/3) in iPSC-CMs at D30 and D90 with ISO (19/3) and the subsequent inhibition of PDE2 (55/7), PDE3 (8/3), and PDE4 (33/3). PDE inhibitors were used following isoprenaline stimulation (EHNA (10 µM), cilostamide (10 µM), rolipram (10 µM); the percentage change in the FRET ratio was normalised to the maximal cAMP produced upon AC activation with NKH477. Data are presented as mean ± SEM. Significance was determined by one-way ANOVA with Bonferroni’s post-hoc test. *** *p* < 0.001. n/N = cells/batches. T = significant from D30.

Following stimulation with ISO, the early cardiomyocytes on D30 displayed greater cytoplasmic β_2_AR-dependent cAMP production than β_1_AR-dependent, and this was reduced with aging on D90 (Figure 4a, clear bars). 

Following PDE2 inhibition, both the β_1_AR- and β_2_AR- dependent cAMP signals increased but only on D90 (Figure 4a,b), which suggests the involvement of this PDE only at later stages of maturation. 

Interestingly, PDE3 inhibition decreased the β_1_AR-dependent cAMP signal in both early and late cardiomyocytes. This shows that this PDE opposes, perhaps indirectly, the activity of other PDEs. Again, the PDE3 inhibition decreased β_2_AR-dependent cAMP signal on D30, whereas it increased this signal at D90 (Figure 4b). Therefore, at a later stage of maturation the involvement of PDE3 in cAMP signalling changes dramatically. 

In contrast, the PDE4 inhibition always gave a strong increase in both the β_1_AR- and β_2_AR-dependent cAMP signal; this effect was seen in all conditions (Figure 4a,b). PDE4 seems to be clearly the main driver for controlling βAR-dependent cAMP production. Interestingly, PDE4 inhibition raised the cAMP production following the stimulation of both βARs higher than did IBMX (a non-specific PDE inhibitor). This suggests some of the phosphodiesterases were having opposing effects on βAR-cAMP, possibly through an indirect effect via cGMP. 

### 3.6. Caveolar Microdomains Compartmentalise β_2_ar and Localise Its Associated Camp Signals

We next investigated whether the PDEs, which are associated with regulating either β_1_AR or β_2_AR-dependent cAMP production, function within caveolae compartments. The disruption of caveolae via cholesterol removal with MβCD was used to assess the association of βAR-dependent cAMP production and PDEs with caveolae. 

**Figure 5:**
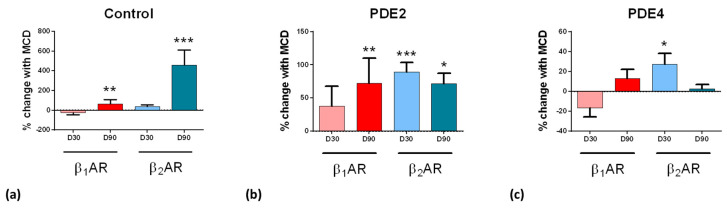
The dependence of PDE2 and 4 activities towards β_1_AR and β_2_AR-stimulated cAMP level on the removal of cholesterol in D30 and D90 iPSC-CMs. (**a**) Difference in the normalised FRET ratio with and without cholesterol removal and subsequent stimulation with isoprenaline (30 nM) in the presence of ICI (50 nM) or CGP (100 nM), respectively, in iPSC-CMs at D30 and D90; (62/11), (55/5), (28/3), (51/3). (**b**) Same with the inhibition of PDE2 with EHNA (10 µM) (15/3), (9/3), (9/3), (8/3); (**c**) Same with inhibition of PDE4 with rolipram (10 µM) (28/4), (23/3), (17/3), (40/3). The percentage change in the FRET ratio was normalised to the maximal cAMP produced upon AC activation with NKH (10 µM). Data are presented as mean ± SEM. Significance was determined by One-Way ANOVA with Bonferroni’s post-hoc test. * *p* < 0.05, ** *p* < 0.01, *** *p* < 0.001, n/N = cells/batches.

Without any inhibition of PDEs, caveolae disruption with MβCD had little effect at D30, but had a strong effect at D90, increasing both the β_1_AR- and β_2_AR-dependent cAMP production; in particular, this effect was stronger for the β_2_AR-dependent cAMP production (Figure 5a). This indicates that β_1_AR- and, to a greater extent, β_2_AR- cAMP signals were associated with caveolae in aged cells. Caveolae removal had a similar effect to IBMX treatment, increasing the β_2_AR-cAMP to comparable levels at D30 and D90. This indicates that both compartmentations through caveolae microdomains or PDE activity play critical roles in compartmentalising cAMP in iPSC-CMs. 

Following the caveolae removal by MβCD treatment, the PDE2 inhibition had a further effect, increasing the cAMP production after both β_1_AR and β_2_AR stimulation in cells of all ages (Figure 5b). This synergistic effect of PDE2 inhibition with MβCD would be consistent with PDE2 activity being separate from caveolae, since it is enhanced, rather than abolished, by caveolar removal. 

With PDE4 inhibition, because the effect of rolipram was so marked, it was difficult to determine whether MβCD was able to enhance it further, with a significant effect only in β_2_AR-cAMP on D30. (Figure 5c, please note the difference in scale from Figure 5a). We conclude that the level of cAMP degradation by PDE4 remains essentially unaltered after caveolae removal.

## 4. Discussion

Caveolar microdomains are pivotal for compartmentalising β_2_ARs and their associated signalling complex in healthy cardiomyocytes. In adult cardiomyocytes, the removal of caveolae increases the spread of β_2_AR -mediated cAMP and dysregulates various ion channels, progressing to arrhythmogenesis in a way which is similar to the loss of T-Tubules during heart failure [29]. In a mature adult heart, β_2_AR signalling plays a central role in regulating the strength and rate of cardiac contraction and relaxation via the classic signalling molecule 3′-5′-cyclic adenosine monophosphate (cAMP). The cAMP signalling pathway is tightly controlled by phosphodiesterase (PDE) enzymes, which restrict diffusion and/or accelerate the degradation of cAMP within the local environment. In cardiomyocytes, PDE2, PDE3, and PDE4 are the primary enzymes responsible for the existence of cAMP gradients in various subcellular compartments [30].

The presence of these structural microdomains is essential for cardiomyocyte function. Jung et al. indicate that the time in culture of iPSC-CMs can encourage β_2_AR signalling maturation [14]. Our study builds on previous knowledge, progressing further into demonstrating that increasing the culture time of iPSC-CMs positively enhances caveolar development and highlighting the existence of a dynamic compartmentation mechanism of the βAR signalling system. Evidence to support this finding comes from an increase in caveolae numbers and a subsequent decrease in the cytoplasmic β_2_AR -cAMP concentration. Focus towards time-dependent maturation events opens an avenue of research in the functional maturation of hiPSC-CMs and similarity to healthy adult cardiomyocytes with respect to the subtype specific expression and the localisation of PDEs in hiPSC-CMs for dictating cAMP compartmentation.

**Figure 6:**
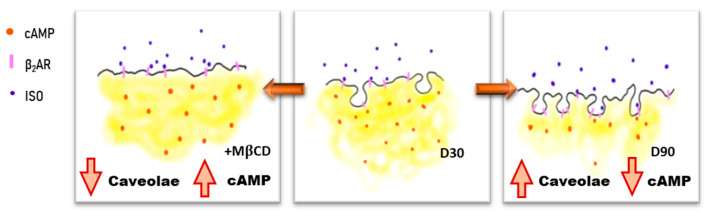
Schematic illustrating the changes in β_2_AR regulation, cAMP level, and caveolae upon the removal of cholesterol and upon the maturation of iPSC-CMs from D30 to D90.

In order to assess the β_2_AR -cAMP generation, attention was given to caveolae and caveolin-3. TEM imaging and gene expression analysis showed that both were expressed likewise in HFKT-iPSC-CM [3], albeit higher in cells at D90 in culture as compared to D30. Treatment with MβCD caused a significant reduction in the numbers of caveolae and a moderate reduction in the caveolin-3 density. Data support the importance of caveolae in maintaining and localising the β_2_AR-cAMP response, reported previously [31]. We find that the disruption of the caveolar microdomains with MβCD caused a significant increase in the β_2_AR-cAMP generation, supporting the idea that caveolae compartmentalise the β_2_AR-cAMP response. Furthermore, increasing the stability of caveolae structures by inducing Cav3 overexpression significantly reduces the β_2_AR-cAMP response (Figure 6). In contrast, minimal changes were seen in the β_1_AR-cAMP response under both conditions, as compared to control. Therefore, our data firmly support the dependency of the localised β_2_AR-cAMP response on stable caveolae microdomains. 

Further downstream of the cAMP signalling cascade, the PDE activity was investigated. IBMX was used as a non-specific PDE inhibitor and signified the importance of PDEs in the the compartmentation of cAMP in hiPSC-CMs by causing a significant increase in the cAMP generation compared to control upon use. The sequentially reduction in the cytoplasmic cAMP in control cells from D30 to D90 indicates the increased compartmentation of the β_2_AR-cAMP response as compared to that of β_1_AR-cAMP over time. This finding supports the hypothesis that β_2_ARs reside in caveolae that increase in numbers with time in culture, further localising the cAMP response. The general inhibition of PDEs with IBMX supports the importance of PDEs in managing βAR-cAMP signalling within the hiPSC-CMs, as found in the adult heart [15,32]. 

Finally, we investigated the influence of specific PDEs on cAMP degradations, using EHNA, Cilostamide, and Rolipram as blockers of PDE2, PDE3, and PDE4, respectively. We find that cAMP generation was highest in the absence of the PDE4 in both receptor subtypes as compared to all other PDE inhibitors tested. These data together show that PDE4—most likely PDE4D—is highly involved with βAR-dependent cAMP compartmentation [33,34,35], with minimal differences in activity over time. The association of PDE cAMP-degrading activity with caveolae was also analysed with MβCD, but the large (possibly maximal) effects of either intervention made it difficult to understand whether effects were additive/synergistic. 

The effects of PDE2 inhibition are still seen on caveolae-depleted cells, suggesting that caveolar location is not essential to PDE2 action. Importantly, PDE2 is activated allosterically by cGMP, such that it enhances PDE2 hydrolytic activity to decrease cAMP production, resulting in negative cGMP-cAMP crosstalk [36]. There are reportedly increased amounts of PDE2 bound to AKAP in failing hearts [37]. Some AKAP families may lie outside caveolae, which may explain the independent effect of PDE2 inhibition. Whichever domain the PDE2 occupies, it does not appear to be strongly affected by the time in culture. 

The inhibition of PDE3 activity seems to inhibit β_1_AR responses at both earlier and late cultured iPS-CMs, and β_2_AR response at day 30. We believe this may be a consequence of PDE crosstalk. PDE3 breaks down both cAMP and cGMP, so there is the possibility of a local cGMP rise with the PDE3 inhibitor. PDE2 can also hydrolyse both cyclic nucleotides, and the binding of cGMP to the regulatory GAF-B domain will increase cAMP affinity and hydrolysis. There is therefore a known feedback loop between the two PDEs that could account for the inhibition of PDE3 decreasing cAMP under certain conditions [38,39].

Our conclusions are in agreement with recent studies which put iPS-CM, even if matured for long time, in the late foetal/early neonatal stages, as compared using modern approaches of single cell genomic analysis of transcriptome [40,41]. 

Collectively, our data shows that early in cardiac differentiation of iPSC-CMs, the βAR signalling system remains relatively immature. However, even at the later stage of maturity, after prolonged culture, the cells’ phenotype does not correspond to mature myocytes, both functionally and morphologically. This can raise concerns for studies using cells at this stage of differentiation as potentially critical pathologic signalling events may be missed. Similarly, studies investigating the drug toxicity and efficacy in early iPSC-CMs may underestimate or miscalculate the response of the postnatal human heart. We report here a strong association between increasing numbers of caveolae with time in culture and the significance this has in localising the β_2_AR -cAMP response in hiPSC-CMs. Our data signify the importance of PDE4 in controlling βAR-dependent cAMP production. 

## Supplementary Materials

The following are available online at https://www.mdpi.com/2073-4409/9/10/2275/s1, Figure S1: Cyclic AMP levels in iCell cell line following cholesterol removal and selective β_1_AR or β_2_AR stimulation. Table S1. Sequences of primers used in the study.
