# Supplementary material for: Age-Dependent Maturation of iPSC-CMs Leads to the Enhanced Compartmentation of β2AR-cAMP Signalling
Source: Cells. 2020 Oct 12;9(10):2275. doi: 10.3390/cells9102275 (PMC7601768; doi:10.3390/cells9102275)
Supplement: Supplementary file 1 [file cells-09-02275-s001.pdf]

Supplementary figure.

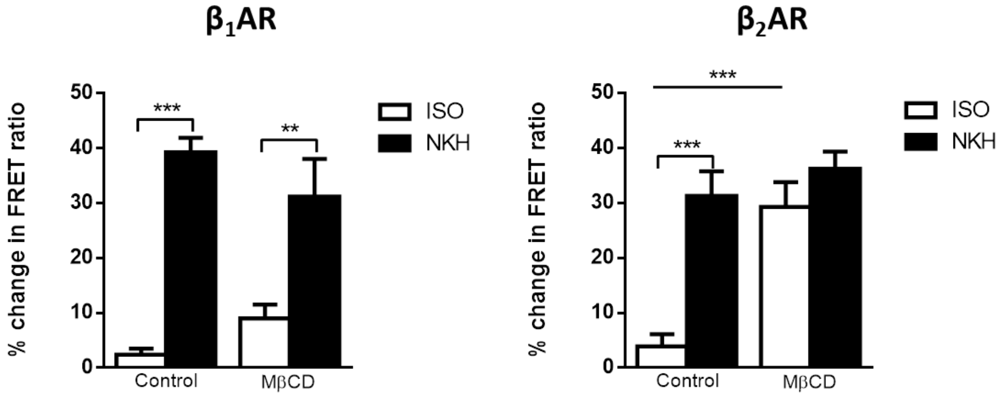

Figure S1. Cyclic AMP levels in iCell cell line following cholesterol removal and selective β1AR or β2AR stimulation.The association of β1AR- (n = 19/3) and β2AR-cAMP (n = 18/5) activity with caveolae was assessed by stimulation with isoprenaline in the presence of ICI or CGP, respectively, with subsequent AC activation with NKH. MβCD was used to deplete caveolae in order to depict the role of caveolae in compartmentalising the β1AR (n = 6/2) and β2AR (n = 9/3) response. Data are presented as mean ± SEM. *p < 0.05, **p < 0.01, ***p < 0.001. n = cells/batches.

Supplementary table

Table S1. Sequences of primers used in the study.

Target Gene	Forward sequence	Reverse sequence
β_1AR	GACGCTCACCAACCTCTTCA	CACAGCTCGCAGAAGAAGGA
β_2AR	TTGCCTCTTCCATCGTGTCC	CCACCTGGCTAAGGTTCTGG
CAV3	GCGGAAGGAGGTCTAAAGC	AGAGCGAAGGGCCATTGT
GAPDH	AATCCCATCACCATCTTCCA	TGGACTCCACGACGTACTCA
